# A case of methotrexate-related lymphoproliferative disease showing multiple liver lesions in a patient with rheumatoid arthritis

**DOI:** 10.1007/s12328-024-01963-6

**Published:** 2024-04-20

**Authors:** Yamato Nagata, Shotaro Akiba, Hidekazu Horiuchi, Kazuo Okumoto, Shigemi Hachinohe, Rintaro Ohe

**Affiliations:** 1 Department of Gastroenterology, Yamagata Prefectural Shinjo Hospital, 720-1 Kanazawa, Shinjo, Yamagata 996-8585 Japan; 2 Department of Pathology, Faculty of Medicine, Yamagata University, Yamagata, Japan

**Keywords:** Liver, Colon, Methotrexate-related lymphoproliferative disease, Diffuse large B-cell lymphoma, Rheumatoid arthritis

## Abstract

A 66-year-old woman with rheumatoid arthritis (RA) who had been receiving methotrexate (MTX) for 2 years presented with tarry stools. Contrast-enhanced computed tomography (CT) of the abdomen revealed irregular wall thickening in the ileocecal region and multiple low-contrast masses in both lobes of the liver. Lower gastrointestinal endoscopy revealed a type 2 tumor in the ileocecal region with a semi-peripheral ulcer. Histological examination of liver and colon biopsies showed other iatrogenic immunodeficiency-associated lymphoproliferative disorder (Oi-LPD), diffuse large B-cell lymphoma type, with positivity for Epstein-Barr virus DNA. After withdrawal of MTX, the LPD lesions disappeared and the patient achieved remission. We considered this to be a sporadic case of Oi-LPD, diffuse large B-cell lymphoma type, in the liver and colon due to treatment with MTX. There has been no previous report of this condition with simultaneous hepatic and colonic lesions, and the present case is thought to be highly informative in relation to the pathogenesis.

## Introduction

Lymphoproliferative disorders (LPDs) arise in patients who have been treated with immunosuppressants, and diffuse large B-cell lymphoma (DLBCL) is the most common type. One such immunosuppressant, methotrexate (MTX), is the first choice for patients with rheumatoid arthritis (RA) [1, 2]. EBV reactivation associated with reduced immunocompetence is known to be responsible for the development of MTX-LPD. As MTX administration for RA tends to be prolonged, development of MTX-LPD is known to be a severe complication [3]. Here we report our experience with a RA patient who developed MTX-LPD, DLBCL type, in the liver and colon.

## Case presentation

**Fig. 1:**
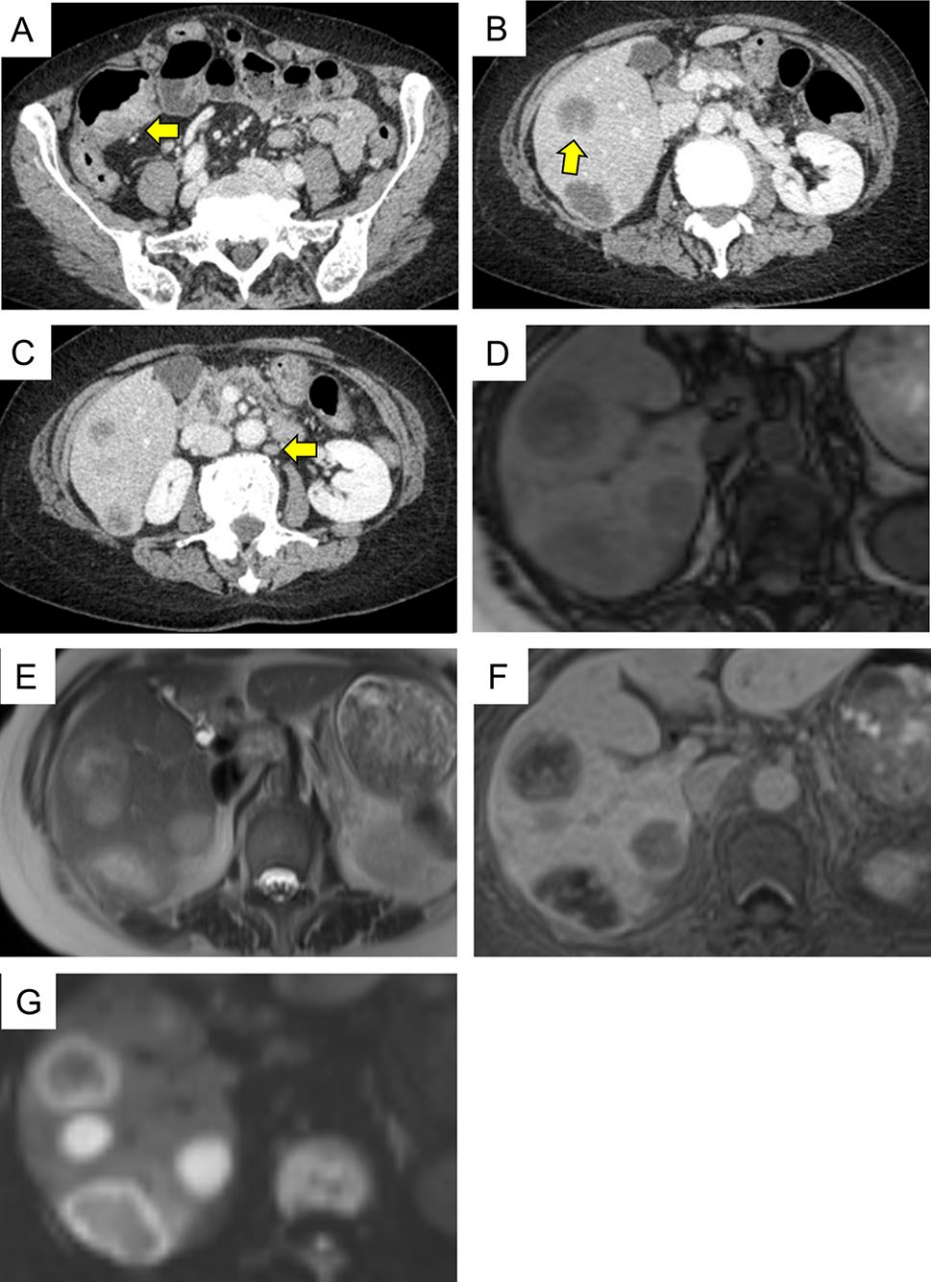
Abdominal imaging modalities. Contrast-enhanced computed tomography (CT) showed a mass with enhancement in the ileocecal region (**A**, *arrow*), and multiple nodular masses without enhancement in the liver (**B**) and para-aortic region (**C**, *arrow*). Penetrating vessels running through the tumor were detected in the liver (**B**, arrow). Contrast-enhanced magnetic resonance imaging (MRI) with gadolinium-ethoxybenzyl diethylenetriamine-penta-acetic acid (Gd-EOB-DTPA) showed that multiple tumors in the liver were lower contrast than the liver parenchyma in portal phase (**D**). T1-weighted images showed low signal in all lesions (**E**). The coarse lesion was centrally high signal on T2-weighted images (**F**) and low signal on DWI images (**G**)

**Fig. 2:**
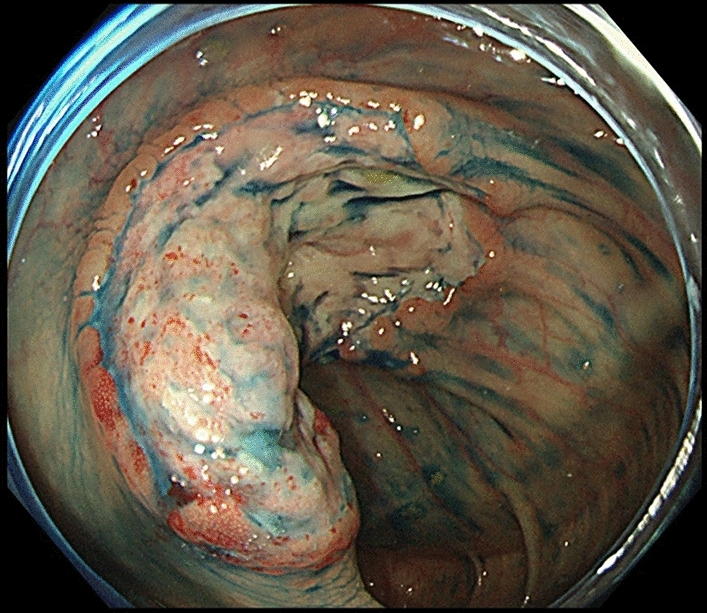
Lower gastrointestinal endoscopy. Lower gastrointestinal endoscopy revealed a mass in the ileocecal region which was suspected malignant tumor

**Fig. 3:**
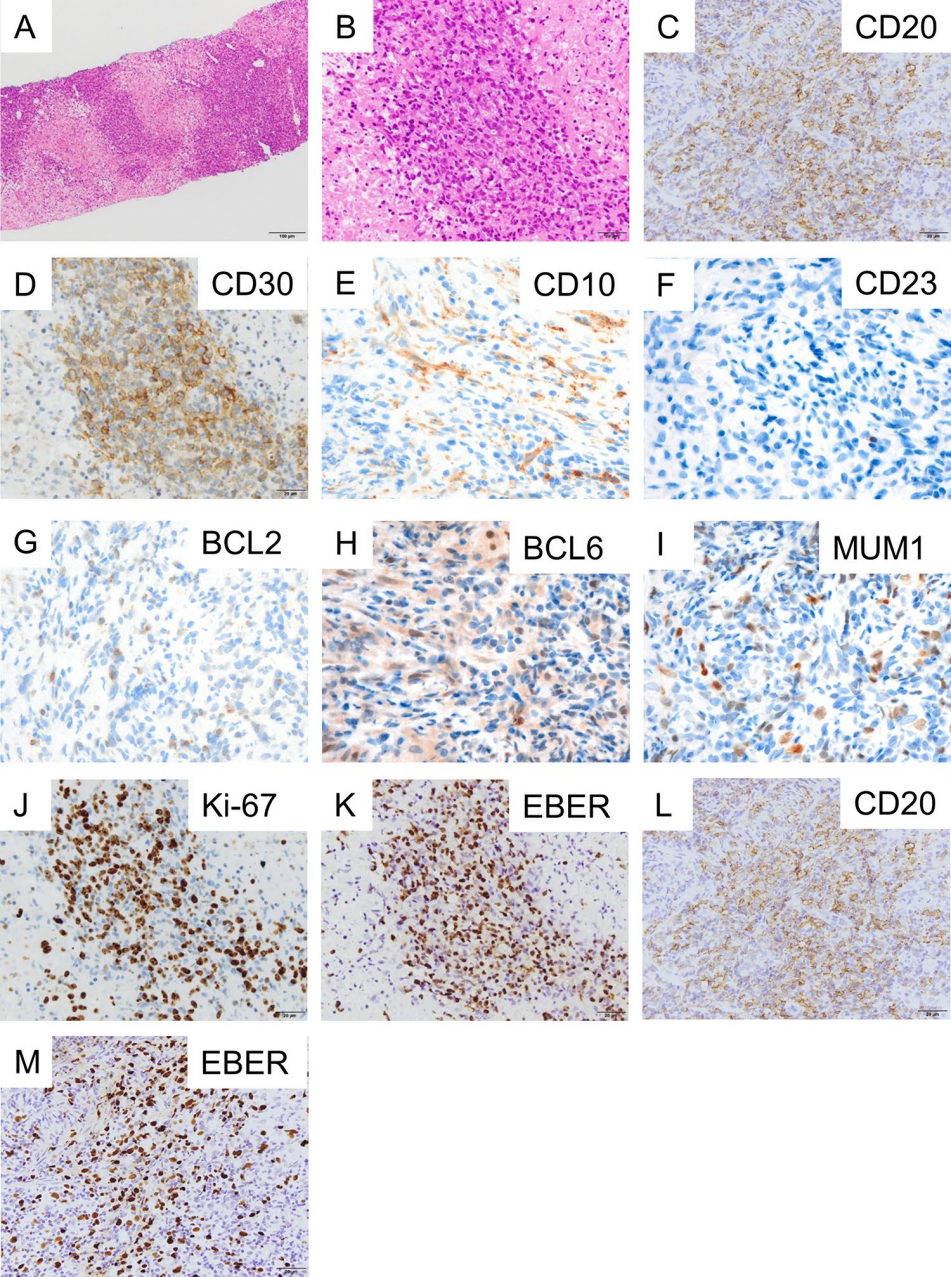
Histological findings in the liver and colon specimens. Histological examination showed diffuse proliferation of mostly large lymphoid cells with necrosis (**A**, **B**). Immunohistochemically, the liver specimens revealed CD20 ( +) (**C**), CD30 ( +) (**D**), CD10 ( +) (**E**), CD 23 (−) (F), BCL2 (−) (G), BCL6 (−) (H), MUM1 (−) (**I**), and Ki-67 labeling index 80% (**J**). EBV encoding RNA (EBER) positivity was detected by in situ hybridization (ISH) (**K**). Similarly, histology of the colon specimens also revealed CD20 ( +) (**L**) and EBER-ISH positivity (**M**)

A 66 year-old woman was admitted to our hospital with a 3 day history of tarry stools. She had RA and had been treated with salazosulfapyridine, iguratimod and MTX for 2 years. Her RA symptoms were well controlled. Her abdomen was flat, soft, and not tender. Severe anemia (hemoglobin 7.6 g/dL) was evident, and the serum levels of C-reactive protein and soluble interleukin-2 receptor were 9.19 mg/dL and 2,630 U/mL, respectively. Epstein–Barr virus (EBV) DNA was detected in the peripheral blood at 3.60 log IU/ml (Table 1). Contrast-enhanced CT revealed a mass with enhancement in the ileocecal region (Fig. 1A), and multiple nodular masses without enhancement in the liver and para-aortic region (Fig. 1B and C). Contrast-enhanced magnetic resonance imaging (MRI) with gadolinium-ethoxybenzyl diethylenetriamine-pentaacetic acid (Gd-EOB-DTPA) showed multiple tumors in the liver with central necrosis (Fig. 1D-G). Lower gastrointestinal endoscopy revealed a mass in the ileocecal region which was suspected malignant tumor (Fig. 2). Liver metastasis of ileocecal cancer or malignant lymphoma was suspected, and ultrasound-guided biopsy of the liver tumors was performed. Histological examination of the liver and colon specimens revealed proliferation of CD20-positive large lymphoid cells with necrosis and in situ hybridization (ISH) demonstrated Epstein-Barr encoding region (EBER)-1 positivity. Histological examination showed diffuse proliferation of mostly large lymphoid cells with necrosis in the liver (Fig. 3A and B) and colon, although there was no atypia in the epithelium of the surface mucosa of colon. Immunohistochemically, these large lymphoid cells were CD20 ( +) (Fig. 3C), CD30 ( +) (Fig. 3D), CD10 ( +) (Fig. 3E), CD 23 (−) (Fig. 3F), BCL2 (−) (Fig. 3G), BCL6 (−) (Fig. 3H), and MUM1 (−) (Fig. 3I). The Ki-67 labeling index was 80% (Fig. 3J). EBER positivity was detected by ISH (Fig. 3K). Immunostaining of biopsies from the colon yielded similar findings (Fig. 3L and M). The final diagnosis was Oi-DLBCL based on the WHO classification revised fourth edition, as the multiple tumors began to shrink after MTX withdrawal.

**Table 1:** Laboratory data of the present case at the time of hospitalization

Peripheral blood		Immunology	
WBC	4880/μL	CRP	9.19 mg/dL
RBC	2.82 × 10^6^/µL		
Hb	7.6 g/dL	Virus markers	
PLT	21.8 × 10^4^/µL	HBsAg	–
Blood coagulation		HCVAb	–
PT (%)	81.0%		
APTT	49.7 s	Tumor markers	
Blood chemistry		CEA	0.438 ng/mL
Alb	2.8 g/dL	CA19-9	4.88 U/mL
BUN	9.8 mg/dL	AFP	2.37 ng/mL
Crea	0.55 mg/dL	PIVKA-II	16 (ECLIA)
T-Bil	0.97 mg/dL	sIL-2R	2630 U/mL
D-Bil	0.31 mg/dL		
AST	17 IU/L	EBV-DNA	3.60 log IU/mL
ALT	13 IU/L		
ChE	155 IU/L		
LDH	223 IU/L		
ALP	114 IU/L		
γ-GTP	25 IU/L		
Na	138 mEq/L		
K	4.8 mEq/L		
Cl	107 mEq/L		
FBS	67 mg/dL		
HbA1c	5.9%		

*PIVKA-II* protein induced by Vitamin K absence or antagonist, *sIL-2R* soluble interleukin-2 receptor

**Fig. 4:**
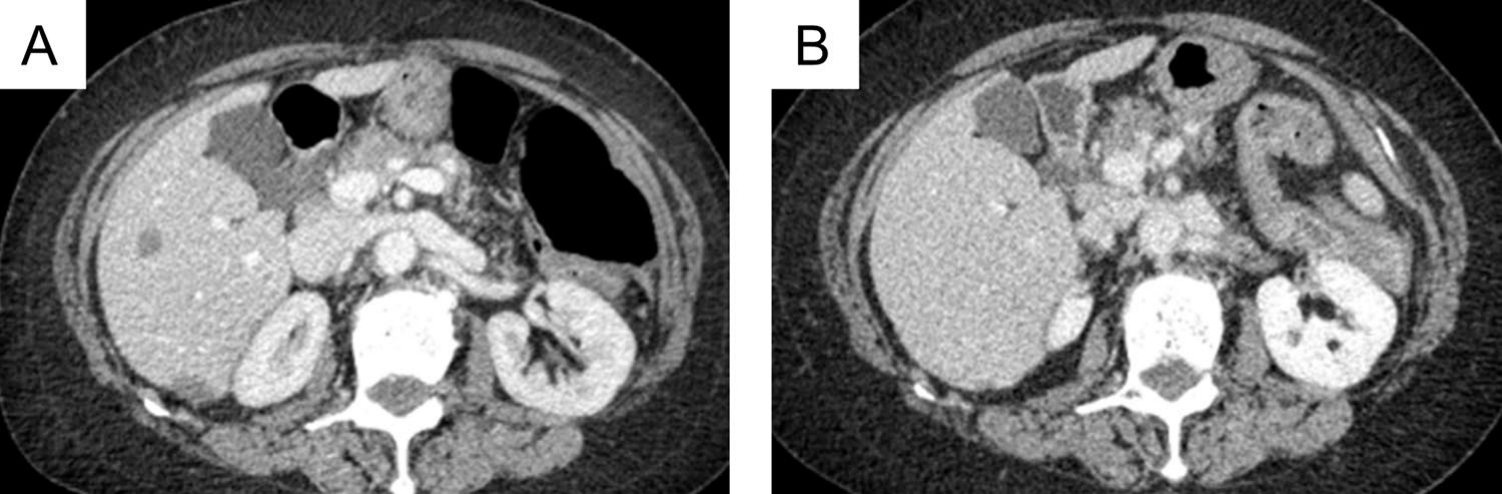
Imaging of the abdomen after methotrexate withdrawal. CT of the abdomen four months later revealed spontaneous shrinkage of most of the tumors (**A**). There has been no sign of recurrence for eight months (**B**)

**Fig. 5:**
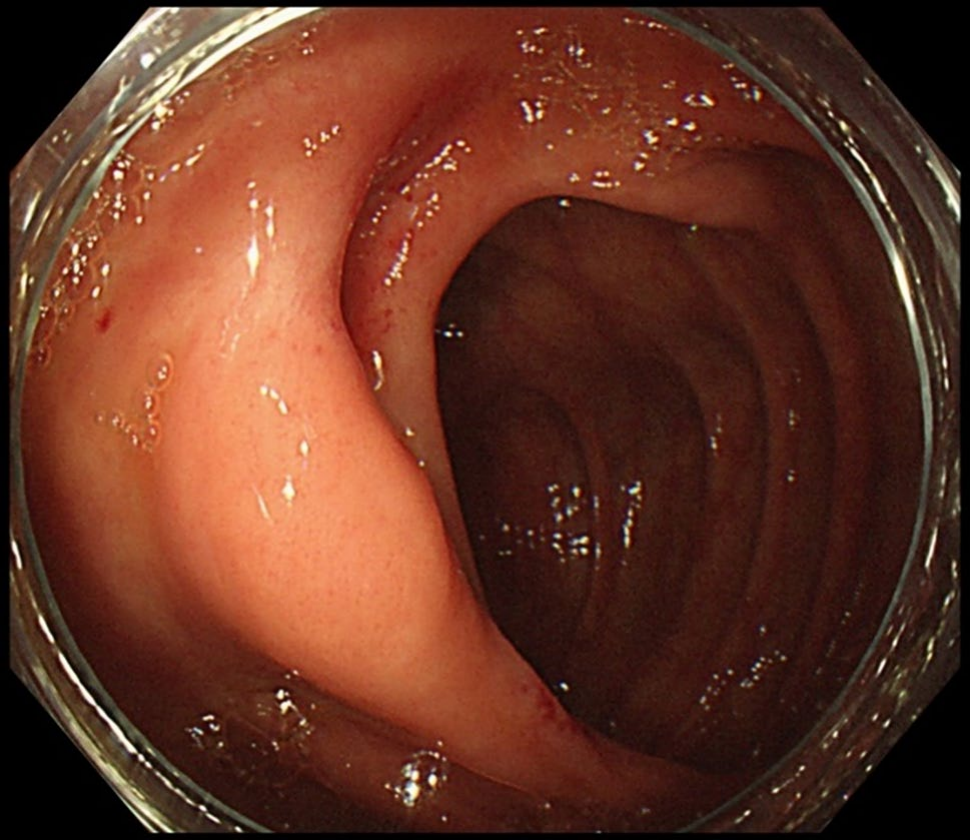
Lower gastrointestinal endoscopy after methotrexate withdrawal. The tumor in the ileocecal region disappeared 8 months later

Observation was continued, and CT of the abdomen four months later revealed spontaneous shrinkage of most of the tumors (Fig. 4A). The serum level of sIL-2R improved spontaneously (312 IU/ml), and EBV DNA in the peripheral blood decreased to less than the standard value. After the withdrawal of MTX, the patient experienced no deterioration in her joints. There has been no sign of recurrence for 8 months (Fig. 4B and 5).

## Discussion

RA patients are known to have a 2- to 4-fold higher incidence of malignant lymphoma as a complication than the general population [4]. Although MTX is used as a first-line treatment for patients with RA, it carries a risk of LPD [2]. When LPD occurs, biologics are often used in the RA patients as an alternative to MTX [5]. LPD, including malignant lymphoma, has been found to occur in RA patients receiving MTX, and this is termed MTX-LPD. The WHO classifies it as immunodeficiency-associated LPD. EBV infection is said to be a complication in about half of MTX-LPD cases, and EBV reactivation has been implicated [6–8]. It is thought that the EBV gene releases factors similar to growth factors, transcription factors, and apoptosis inhibitors, causing B lymphocytes to transform into lymphoblasts, exhibiting tumor-like growth [9]. The most common sites of MTX-LPD are lymph nodes [10] and tonsils [11], but thyroid [12], lung [13], liver [14], and colon lesions [15] have also been reported.

Imaging modalities, such as US and CT, can aid the diagnosis of hepatic lymphoma/MTX-LPD. A previous report has indicated that penetrating vessels running through the tumor visualized by US was helpful for diagnosis of hepatic lymphoma [16]. In the present case, penetrating vessels running through the tumor were detected by CT scan. The evidence suggests that MTX-LPD should be considered in RA patients who are receiving MTX medications when CT or US shows a hepatic mass with signs of penetrating vessels.

A search of PubMed using the terms “MTX-LPD” and “hepatic”, or “MTX-LPD” and “liver”, yielded 12 available studies published in English between 2000 and 2022 [10, 14, 17–26]. In all cases the patients had been treated with immunosuppressants such as MTX, steroids, infliximab, and tacrolimus. All had taken MTX for more than 24 months, and the median total dose of MTX was 1932 mg (960–6000 mg). Ten patients showed reactivation of EBV. The present patient had taken MTX for 24 months (total dose, 960 mg) and also showed reactivation of EBV. MTX-LPD involving the liver along with other parenchymal organs must be differentiated from cancer metastasis. As shown in Table 2, five patients with hepatic MTX-LPD accompanied by other parenchymal organ lesions involvement of the adrenal gland (*n* = 2), multiple lymph nodes (*n* = 1), spleen (*n* = 1), and colon (*n* = 1, this case).

**Table 2:** Reported cases of rheumatoid arthritis patients with methotrexate-related lymphoproliferative disease in the liver

Case	Year	Age, Sex	Total MTX dose (mg)	Other drugs	Pathology	Others	EBER	Imaging modalities	Treatment	Outcome
1	Soubrier, et al. 2006	48, F	1268	None	DLBCL	None	positive	CT	Discontinuation of MTX	Remission
2	Fujita, et al. 2013	69, M	ND	None	Lymphocytes and interstinal fibrosis	None	positive	CT, MRI, PET	Discontinuation of MTX	Remission
3	Tatsumi, et al. 2014	67, F	1872	PSL	DLBCL	None	positive	CT, US	Discontinuation of MTX R-THP-COP 6 courses	Remission
4	Kawahara, et al. 2015	64, M	1056	IFX	DLBCL	None	negative	CT, US	Discontinuation of MTX R-CHOP 8 courses	Remission
5	Miyagawa, et al. 2015	56, F	5304	None	DLBCL	None	negative	CT, US	Discontinuation of MTX R-CHOP 6 courses	Remission
6	Ohkura, et al. 2015	70, M	5004	None	HL	Adrenal grand	positive	CT, MRI	Hepatectomy Discontinuation of MTX	No recurrence
7	Matsumoto, et al. 2016	63, M	3110	None	DLBCL	Adrenal grand	positive	US, CT, PET	Discontinuation of MTX	Remission
8	Takei, et al. 2017	65, F	ND	None	DLBCL	None	positive	CT, MRI, US	Hepatectomy Discontinuation of MTX	No recurrence
9	Tsukazaki, et al. 2017	88, F	1932	IFX	HL	Lymph nodes	positive	CT	Discontinuation of MTX Chemotherapy	Recurrence but remission
10	Ono, et al. 2018	82, M	3454	PSL,BUC	B cell lymphoma	Spleen & lymph nodes	negative	CT	Discontinuation of MTX	Remission
11	Omameuda, et al. 2022	64, F	6000	BUC,PSL	DLBCL	None	positive	CT, MRI	Hepatectomy Discontinuation of MTX	No recurrence
12	Ito, et al. 2022	72, F	1728	IGU	ND	None	EBV-DNA positive	CT	Discontinuation of MTX	No recurrence
13	This case	66, F	960	SASP,IGU	DLBCL	Colon & lymph nodes	positive	CT, MRI, US	Discontinuation of MTX	No recurrence

*MTX* methotrexate, *EBER* Epstein–Barr virus-encoded small ribonucleic acid, *F* female, *M* male, *DLBCL* diffuse large B cell lymphoma, *HL* Hodgkin lymphoma, *CT* computed tomography, *MRI* magnetic resonance *PET* positron emission tomography, *US* ultrasonography, *PSL* prednisolone, *IFX* infliximab, *BUC* bucillamine, *IGU* iguratimod, *SASP* salazosulfapyridine, *R-THP-COP* rituximab, cyclophosphamide, pirarubicin, and vincristine/prednisolone, *R-CHOP* rituximab, adriamycin, cyclophosphamide, vincristine, and prednisolone, *ND* no data

In the listed MTX-LPD, DLBCL type cases (*n* = 8), MTX was discontinued and three of the patients achieved complete remission. Three additional patients achieved complete remission with chemotherapy. Two patients were treated by partial hepatectomy and had no recurrence. Two patients with MTX-LPD, Hodgkin lymphoma type, also stopped using MTX. One of them suffered relapse even after withdrawal of MTX, and the other underwent partial hepatectomy and had no recurrence. Our present patient achieved complete remission after MTX had been withdrawn. These findings suggest that immediate withdrawal of MTX is recommended for patients with hepatic MTX-LPD, DLBCL type. Our patient had two lesions; these may have been concurrent, or one may have been due to metastasis from the colon to the liver. Because of the multiple lymph node metastases and the histological similarities of the lesions, the possibility that the colon lesion had metastasized to the liver could not be ruled out. To our knowledge, this is the first case of MTX-LPD involving both the liver and colon to have been reported in English.

## Conclusion

We have experienced a case of MTX-LPD, DLBCL type, in which multiple liver masses and colon lesions were concurrent. Multiple systemic masses in RA patients receiving MTX are suggestive of MTX-LPD, and early discontinuation of MTX should be considered.

## References

[1] Singh JA, Saag KG, Bridges SL Jr, et al. 2015 American college of rheumatology guideline for the treatment of rheumatoid arthritis. Arthritis Rheumatol. 2016;68:1–26. 26545940 10.1002/art.39480

[2] Ohe R, Yang S, Yamashita D, et al. Pathogenesis of follicular thymic hyperplasia associated with rheumatoid arthritis. Pathol Int. 2022;72:252–60. 35147259 10.1111/pin.13212 PMC9304286

[3] Saito S, Takeuchi T. Immune response in LPD during methotrexate administration (MTX-LPD) in rheumatoid arthritis patients. J Clin Exp Hematop. 2019;59:145–55. 31866617 10.3960/jslrt.19028 PMC6954173

[4] Anderson LA, Gadalla S, Morton LM, et al. Population-based study of autoimmune conditions and the risk of specific lymphoid malignancies. Int J Cancer. 2009;125:398–405. 19365835 10.1002/ijc.24287 PMC2692814

[5] Katsuyama T, Sada KE, Yan M, et al. Prognostic factors of methotrexate-associated lymphoproliferative disorders associated with rheumatoid arthritis and plausible application of biological agents. Mod Rheumatol. 2017;27:773–7. 27846761 10.1080/14397595.2016.1259714

[6] Kikuchi K, Miyazaki Y, Tanaka A, et al. Methotrexate-related epstein-barr virus (EBV)-associated lymphoproliferative disorder–so-called “Hodgkin-like lesion”–of the oral cavity in a patient with rheumatoid arthritis. Head Neck Pathol. 2010;4:305–11. 20676828 10.1007/s12105-010-0202-6 PMC2996501

[7] Kitamura N, Sugiyama K, Nagasawa Y, et al. Involvement of Epstein-Barr virus in the development and spontaneous regression of methotrexate-associated lymphoproliferative disorder in patients with rheumatoid arthritis. Clin Exp Rheumatol. 2022;40:1330–5. 34369356 10.55563/clinexprheumatol/lgfbtq

[8] Afonso C, Roque A, Almeida C, et al. Methotrexate-associated lymphoproliferative disorder in a patient with psoriasis: a case report and review of the literature. Case Rep Hematol. 2022;2022:7178065. 35535243 10.1155/2022/7178065 PMC9078817

[9] Grywalska E, Markowicz J, Grabarczyk P, et al. Epstein-Barr virus-associated lymphoproliferative disorders. Postepy Hig Med Dosw (Online). 2013;67:481–90. 23752600 10.5604/17322693.1050999

[10] Tsukazaki Y, Shinohara T, Tanaka K, et al. Hepatosplenic Hodgkin lymphoma without lymphadenopathy following reversible methotrexate-associated lymphoproliferative disorder. Mod Rheumatol. 2017;27:372–5. 25401225 10.3109/14397595.2014.977513

[11] Watanabe T, Teratani Y. Unusual manifestation of methotrexate-associated lymphoproliferative disorder as a palatal mass. BMJ Case Rep. 2022. 10.1136/bcr-2022-250616. 36175042 10.1136/bcr-2022-250616 PMC9528599

[12] Hiruma M, Tsuboi K, Hirose T. Methotrexate-associated lymphoproliferative disorder in the thyroid gland of a patient with chronic thyroiditis. J Med Ultrason. 2001;2023(50):575–6. 10.1007/s10396-023-01344-5 37526779

[13] Suemori K, Hasegawa H, Ishizaki J, et al. Methotrexate-associated lymphoproliferative disease with multiple pulmonary nodules in a patient with rheumatoid arthritis. Intern Med. 2015;54:1421–5. 26028000 10.2169/internalmedicine.54.3542

[14] Omameuda T, Miyato H, Sata N, et al. Primary hepatic methotrexate-associated lymphoproliferative disorder associated with Epstein-Barr virus reactivation and accompanied by spontaneous necrosis: a case report. Medicine (Baltimore). 2022;101: e31993. 36451467 10.1097/MD.0000000000031993 PMC9705001

[15] Shirakabe K, Mizokami K. Methotrexate-associated lymphoproliferative disease detected as a colorectal mass lesions: a case report. J Surg Case Rep. 2023. 10.1093/jscr/rjad098. 36896165 10.1093/jscr/rjad098 PMC9991593

[16] Takasumi M, Okai K, Asano T, et al. A case of methotrexate-associated lymphoproliferative disorder diagnosed by liver biopsy. Nihon Shokakibyo Gakkai Zasshi. 2015;112(1):115–22. 25744928 10.11405/nisshoshi.112.115

[17] Soubrier M, Arrestier S, Bouloudian S, et al. Epstein-Barr virus infection associated hepatic lymphoma in a patient treated with methotrexate for rheumatoid arthritis. Joint Bone Spine. 2006;73:218–9. 16495108 10.1016/j.jbspin.2005.06.005

[18] Fujita T, Tanabe M, Iida E, et al. Multi-modality imaging findings of methotrexate-related Epstein-Barr virus-associated hepatic tumor. Clin Imaging. 2013;37:962–4. 23849101 10.1016/j.clinimag.2013.05.005

[19] Tatsumi G, Ukyo N, Hirata H, et al. Primary hepatic lymphoma in a patient with rheumatoid arthritis treated with methotrexate. Case Rep Hematol. 2014;2014: 460574. 25610674 10.1155/2014/460574 PMC4290657

[20] Kawahara A, Tsukada J, Yamaguchi T, et al. Reversible methotrexate-associated lymphoma of the liver in rheumatoid arthritis: a unique case of primary hepatic lymphoma. Biomark Res. 2015;3:10. 25964853 10.1186/s40364-015-0035-2 PMC4426646

[21] Miyagawa K, Shibata M, Noguchi H, et al. Methotrexate-related primary hepatic lymphoma in a patient with rheumatoid arthritis. Intern Med. 2015;54:401–5. 25748956 10.2169/internalmedicine.54.3361

[22] Ohkura Y, Shindoh J, Haruta S, et al. Primary adrenal lymphoma possibly associated with Epstein-Barr virus reactivation due to immunosuppression under methotrexate therapy. Medicine (Baltimore). 2015;94: e1270. 26252293 10.1097/MD.0000000000001270 PMC4616607

[23] Matsumoto R, Numata K, Doba N, et al. A case of multiple hepatic lesions associated with methotrexate-associated lymphoproliferative disorder. J Med Ultrason. 2001;2016(43):545–51. 10.1007/s10396-016-0740-y 27577564

[24] Takei D, Abe T, Amano H, et al. Methotrexate-associated primary hepatic malignant lymphoma following hepatectomy: a case report. Int J Surg Case Rep. 2017;31:5–9. 28076752 10.1016/j.ijscr.2016.12.012 PMC5222945

[25] Ono R, Kumagae T, Uojima H, et al. Hepatic methotrexate-associated lymphoproliferative disorders identified by multiple liver tumors: a case report and review of the literature. J Med Case Rep. 2019;13:196. 31242930 10.1186/s13256-019-2135-3 PMC6595583

[26] Ito N, Masuda T, Yamaguchi K, et al. Pneumonia and meningoencephalitis due to varicella-zoster virus reinfection and Epstein-Barr virus reactivation in a patient with rheumatoid arthritis. Intern Med. 2022;61:2961–5. 35249916 10.2169/internalmedicine.8413-21 PMC9593163

